# The Computational Development of Reinforcement Learning during Adolescence

**DOI:** 10.1371/journal.pcbi.1004953

**Published:** 2016-06-20

**Authors:** Stefano Palminteri, Emma J. Kilford, Giorgio Coricelli, Sarah-Jayne Blakemore

**Affiliations:** 1 Institute of Cognitive Neuroscience, University College London, London, United Kingdom; 2 Laboratoire de Neurosciences Cognitive, École Normale Supérieure, Paris, France; 3 Interdepartmental Centre for Mind/Brain Sciences, Università degli Studi di Trento, Trento, Italy; 4 Department of Economics, University of Southern California, Los Angeles, California, United States of America; Oxford University, UNITED KINGDOM

## Abstract

Adolescence is a period of life characterised by changes in learning and decision-making. Learning and decision-making do not rely on a unitary system, but instead require the coordination of different cognitive processes that can be mathematically formalised as dissociable computational modules. Here, we aimed to trace the developmental time-course of the computational modules responsible for learning from reward or punishment, and learning from counterfactual feedback. Adolescents and adults carried out a novel reinforcement learning paradigm in which participants learned the association between cues and probabilistic outcomes, where the outcomes differed in valence (reward versus punishment) and feedback was either partial or complete (either the outcome of the chosen option only, or the outcomes of both the chosen and unchosen option, were displayed). Computational strategies changed during development: whereas adolescents’ behaviour was better explained by a basic reinforcement learning algorithm, adults’ behaviour integrated increasingly complex computational features, namely a counterfactual learning module (enabling enhanced performance in the presence of complete feedback) and a value contextualisation module (enabling symmetrical reward and punishment learning). Unlike adults, adolescent performance did not benefit from counterfactual (complete) feedback. In addition, while adults learned symmetrically from both reward and punishment, adolescents learned from reward but were less likely to learn from punishment. This tendency to rely on rewards and not to consider alternative consequences of actions might contribute to our understanding of decision-making in adolescence.

## Author Summary

We employed a novel learning task to investigate how adolescents and adults learn from reward versus punishment, and to counterfactual feedback about decisions. Computational analyses revealed that adults and adolescents did not implement the same algorithm to solve the learning task. In contrast to adults, adolescents’ performance did not take into account counterfactual information; adolescents also learned preferentially to seek rewards rather than to avoid punishments, whereas adults learned to seek and avoid both equally. Increasing our understanding of computational changes in reinforcement learning during adolescence may provide insights into adolescent value-based decision-making. Our results might also have implications for education, since they suggest that adolescents benefit more from positive feedback than from negative feedback in learning tasks.

## Introduction

Adolescence is defined as the period of life that starts with the biological changes of puberty and ends with the individual attainment of a stable, independent role in society[1]. During this period, significant changes in value-based decision-making are observed[2]. Adolescents are often characterised as prone to engage in suboptimal decision-making, which although probably adaptive in many circumstances[3–6], can sometimes result in negative real life outcomes[7,8].

The computational framework of reinforcement learning formally captures value-based decision-making[9,10]. Reinforcement learning (RL) refers to the ability to learn to improve one’s future choices in order to maximise the expected value. The simplest RL algorithm (Q-learning) learns action-outcome associations directly from experienced rewards on a trial and error basis[11,12]. However, more complex behaviours, such as counterfactual learning and punishment- avoidance learning cannot be explained using the basic RL algorithm, due to its computational simplicity. Counterfactual learning refers to the ability to learn not only from direct experience, but also from hypothetical outcomes (the outcomes of the option(s) that were not chosen)[13,14]. Punishment avoidance, compared to reward seeking, requires an additional computational step in which outcomes are considered relative to a reference point (i.e. outcome valuation is contextualised)[15,16]. Thus, compared to simple reward seeking, counterfactual and avoidance learning are more computationally demanding. Accordingly, whereas simple reward learning has been largely and robustly associated with the striatum[17–19], punishment and counterfactual processing have been consistently associated with the dorsal prefrontal system and the insula, areas that are classically associated with cognitive control[13,20–23]. Theories of adolescent brain development have pointed to differential functional and anatomical development of limbic regions, such as the striatum, and cognitive control regions and there is some evidence to support this notion [1,2,6,24–26]. We hypothesise that this asymmetrical development might be translated into a difference in the computational strategies used by adolescents compared with adults. Differences in reinforcement learning strategies may in turn contribute to an explanation of features of adolescent value-directed behaviour.

**Fig 1:**
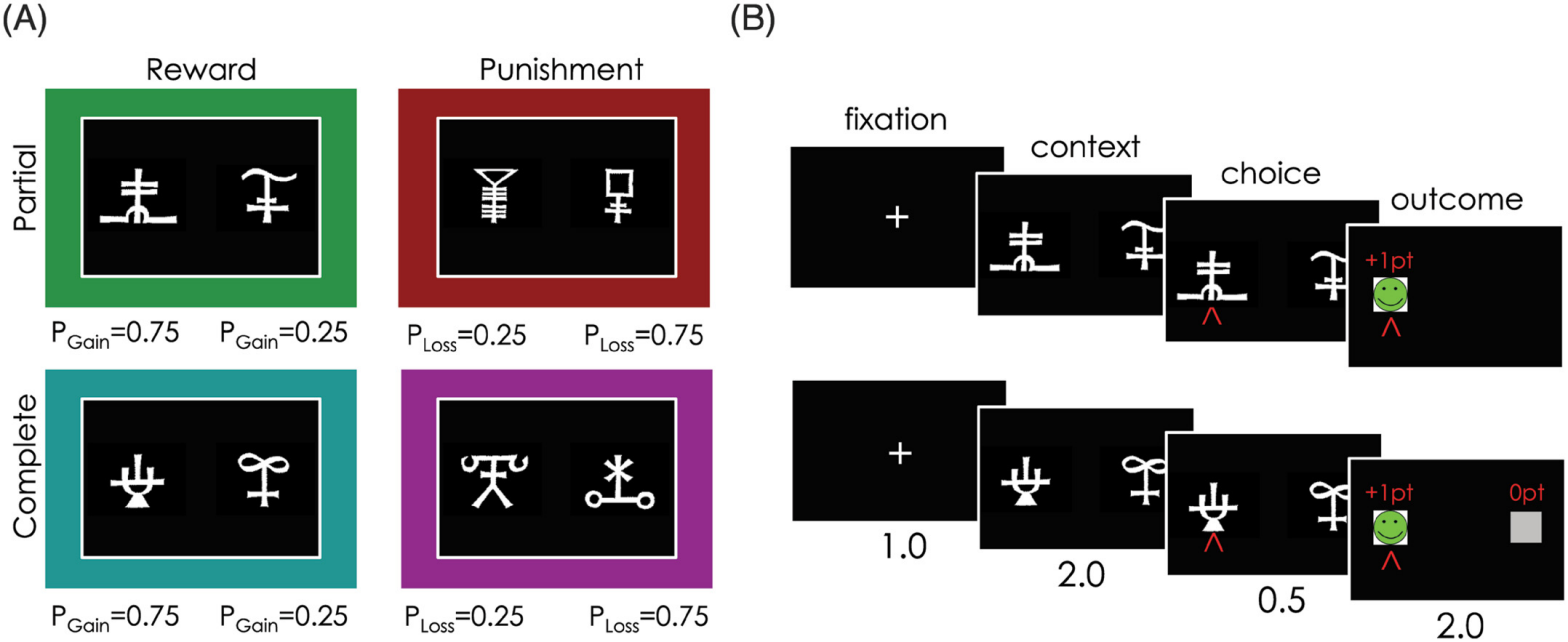
Task design. **(A)** The learning task 2x2 factorial design. Different symbols were used as cues in each context, and symbol to context attribution was randomised across participants. The coloured frames are purely illustrative and represent each of the four context conditions throughout all figures. “Reward” = gain maximisation context; “Punishment” = loss minimisation context; “Partial”: counterfactual feedback was not provided; “Complete”: counterfactual feedback was provided; P_Gain_ = probability of gaining 1 point; P_Loss_ = probability of losing 1 point. **(B)** Time course of example trials in the Reward/Partial (top) and Reward/Complete (bottom) conditions. Durations are given in seconds. Fig 1 was adapted by the authors from a figure originally published in [15], licensed under CC BY 4.0.

**Fig 2:**
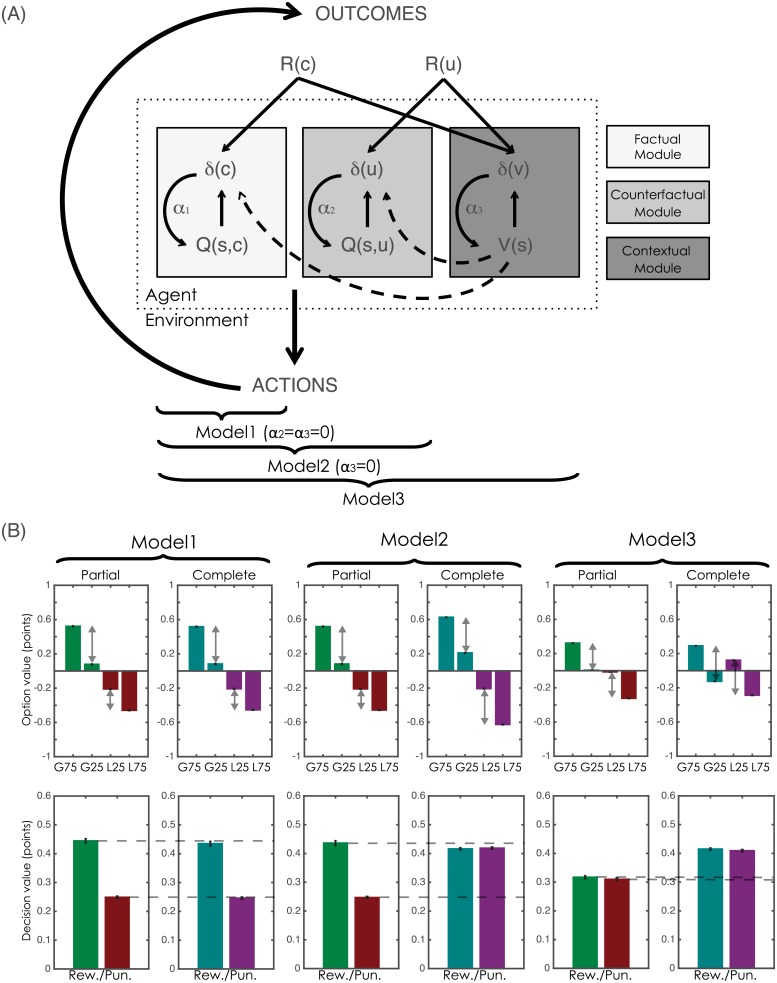
Computational models and ex-ante model simulations. **(A)** The schematic illustrates the computational architecture of the model space. For each context (or state, ‘s’), the agent tracks option values (Q(s,:)), which are used to decide amongst alternative courses of action. In all contexts, the agent is informed about the outcome corresponding to the chosen option (R(c)), which is used to update the chosen option value (Q(s,c)) via a prediction error (δ(c)). This computational module (“factual module”) requires a learning rate (α_1_). In the presence of complete feedback, the agent is also informed about the outcome of the unchosen option (R(u)), which is used to update the unchosen option value (Q(s,u)) via a prediction error (δ(u)). This computational module (“counterfactual module”) requires a specific learning rate (α_2_). In addition to tracking option value, the agent also tracks the value of the context (V(s)), which is also updated via a prediction error (δ(v)), integrating over all available feedback information (R(c) and, where applicable, R(u)). This computational module (“contextual module”) requires a specific learning rate (α_3_). The full model (Model 3) can be reduced to Model 2 by suppressing the contextual module (i.e. assuming α_3_ = 0). Model 2 can be reduced to Model 1 (basic Q-learning) by suppressing the counterfactual module (i.e. assuming α_2_ = α_3_ = 0). (**B**). Bars represent the model estimates of option values (top row) and decision values (bottom row), plotted as a function of the computational models and task contexts. G75 and G25: options associated with 75% and 25% chance of gaining a point, respectively; L75 and L25: options associated with 75% and 25% chance of losing a point, respectively. “Decision value” represents the difference in value between the correct and incorrect options (G75 minus G25 in Reward contexts; L25 minus L75 in Punishment contexts). Fig 2A was adapted by the authors from a figure originally published in [15], licensed under CC BY 4.0.

More precisely, we hypothesise that, while the basic RL algorithm successfully encapsulates value-based decision-making in adolescence, adults integrate more sophisticated computations, such as counterfactual learning and value contextualisation. To test this hypothesis, adults and adolescents performed an instrumental probabilistic learning task in which they had to learn which stimuli had the greatest likelihood of resulting in an advantageous outcome through trial and error. Both outcome valence (Reward vs. Punishment) and feedback type (Partial vs. Complete) were manipulated using a within-subjects factorial design (Fig 1A). This allowed us to investigate both punishment avoidance learning and counterfactual learning within the same paradigm. In a previous study, model comparison showed that adult behaviour in this task is best explained by a computational model in which basic RL is augmented by a counterfactual learning module (to account for learning from outcomes of unchosen options) and a value contextualisation module (to account for learning efficiently to avoid punishments) (Fig 2A)[15]. Our computational and behavioural results are consistent with our hypothesis and show that adolescents utilise a different, simpler computational strategy to perform the task.

## Results

### Computational models

During the learning task, participants made choices between two options, presented within different choice contexts (Fig 1). In each context, one option had a higher probability of resulting in an advantageous outcome (the ‘correct’ option; gaining a point or not losing a point) than the other. We submitted participants’ correct choice rate to computational analyses, based on an algorithm that has been shown to provide a good account for both behavioural and neural data within the same task in adults (Fig 2A)[15]. In short, the model includes a factual learning module (Q-learning), which updates the value of the chosen option (governed by a first free parameter: α_1_), a counterfactual learning module, which updates the value of the unchosen option (governed by a second free parameter: α_2_) and, finally, a contextual learning module, which learns the average value of the choice context and uses this to move from an absolute to a relative encoding of option value (governed by a third free parameter: α_3_). The counterfactual learning module has been shown to underlie the enhanced learning induced by the presence of complete feedback information, whereas the contextual learning model has been proposed to underpin the ability to perform similarly in both punishment and reward contexts. Thus, our model space included three nested and increasingly sophisticated models. Model 1 was a simple, option-value learning model (Q-learning), with no counterfactual or contextual learning modules (α_2_ = α_3_ = 0). Model 2 also included counterfactual, but no contextual learning (α_3_ = 0). Finally, Model 3 was the “complete” model. Model 3 can be seen as the most parsimonious translation into the reinforcement-learning framework of the fictive learning models and relative value-based decision-making models proposed in economics[27,28].

### Ex-ante model simulations: Learning test

To describe the properties of the three models and illustrate how their performances differ across the different choice contexts (states, ‘s’), we ran ex-ante model simulations and analysed the model estimates of option values (Q(s,:)) and decision values (ΔQ(s)) (Fig 2B). Decision value is defined for each context as the difference in value between the correct and incorrect option. Decision values ultimately determine the percentage of correct choices during the learning task. Model 1 (basic Q-learning) predicts higher performance in the Reward compared to the Punishment contexts, a learning asymmetry predicted by the punishment avoidance learning paradox[29], and similar performance in the Partial and Complete feedback contexts. Model 2 (Model 1 plus the counterfactual learning module) permits an improvement in performance in the Punishment/Complete context, however still predicts a learning asymmetry in the Partial feedback contexts. Finally, Model 3 (Model 2 plus the value contextualisation module) predicts similar performance in the Reward and Punishment contexts and increased performance in the Complete compared to the Partial feedback contexts: this is the behavioural pattern that we expected for the adult group, based on our previous study[15].

### Model fitting: Baseline quality of fit does not differ between adolescents and adults

**Fig 3:**
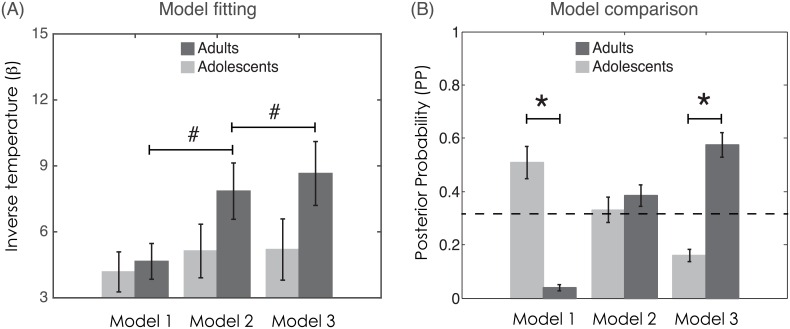
Baseline model fitting and model comparison. **(A)** Choice inverse temperature (β) of each model for adults (dark grey) and adolescents (light grey). (**B**). Posterior probability (PP) of each model for adults (dark grey) and adolescents (light grey). The dotted line indicates chance level (0.33). ^#^P<0.05; 2-sided, one-sample, t-tests; *P<0.001; 2-sided, independent samples, t-tests. Error bars represent s.e.m.

We fitted the three models to individual histories of choices and outcomes, in order to obtain, for each participant and each model, the parameters that maximised the negative log-likelihood of participants’ choices during the learning task (see S1 Table). To assess whether baseline model fitting differed between adolescents and adults, we submitted the negative log-likelihood and the inverse temperature parameter (β) to mixed-design ANOVA with group (Adolescents vs. Adults) as the between-subjects factor and model as the within-subjects factor. For negative log-likelihood (a measure of model quality of fit), there was no main effect of group (F(1,36) = 1.3, P>0.2) and the group x model interaction did not reach significance (F(2,72) = 2.7, P<0.08). Note that the main effect of model cannot be tested since the models are nested and therefore the negative log-likelihood can only decrease. Analysis of the inverse temperature (β) parameter supported these results. This parameter can be taken as a measure of how well choices are predicted by the model and strongly correlates with the model likelihood (for all models: R>0.93; P<0.001). There was no main effect of group (F(1,36) = 2.3, P>0.1) but there was a significant group x model interaction (F(2,72) = 5.0, P<0.01) (Fig 3A). Post-hoc comparisons showed that this interaction was driven by adults showing increases in inverse temperature when comparing Model 1 to Model 2 (T(19) = 3.2, P<0.01) and Model 2 to Model 3 (T(19) = 2.2, P<0.05). Baseline (Model 1) inverse temperature did not differ between adults and adolescents (T(36) = 0.4, P>0.70). The absence of main effects of group indicates that baseline quality of fit was not different between age groups, thus allowing further model comparison analyses.

### Model comparison: Different computational models explain learning in adolescents compared to adults

Posterior probability (PP) was calculated for each of the models, using the log of the Laplace approximation of the model evidence. Similar to other model comparison criteria, quality of fit is penalised by the model complexity[30]. As above, we submitted the PP of the models to a mixed-design ANOVA with group as the between-subjects factor and model as the within-subjects factor (Fig 3B). This analysis indicated a significant group x model interaction (F(2,72) = 38.9, P<0.001). The effect of model was not quite significant (F(2,72) = 3.0, P<0.06). Note that the main effect of group cannot be tested, since the model posterior probabilities by definition must sum to one, thus creating equal group means. Post-hoc comparisons showed that in the adolescent group, the posterior probability of Model 1 was significantly greater than chance level (T(17) = 3.0, P<0.01; exceedance probability = 0.77) and greater than that of the adult group (T(36) = 8.0, P<0.001). Conversely, in adults, the posterior probability of Model 3 was significantly greater than chance level (T(19) = 5.2, P<0.001; exceedance probability = 0.80) and greater than that of the adolescents (T(36) = 7.8, P<0.001) (see also Tables 1, S1, S2 and S3). This result indicates that different computational models explain learning behaviour in the two groups. More precisely, a simple RL model better describes adolescents’ behaviour, whereas a more complex model, which integrates counterfactual and contextual learning processes, better accounts for adults’ behaviour.

**Table 1:** Bayesian Model comparison.

	Model 1		Model 2		Model 3	
	PP	XP	PP	XP	PP	XP
**Subject -level**						
**Adoles.**	0.51±0.06	0.77	0.33±0.05	0.20	0.16±0.02	0.02
**Adults**	0.04±0.01	0.00	0.38±0.04	0.20	0.57±0.05	0.79
**Group-level**						
**Adoles.**	0.70	0.48	0.21	0.28	0.08	0.24
**Adults**	0.00	0.21	0.31	0.32	0.68	0.47

PP: posterior probability. XP: exceedance probability. Subject-level: parameters optimization assuming a set of free parameters per subject. Group-level: parameters optimization assuming a single set of free parameters per age group. PPs are reported as mean±s.e.m.

### Behavioural analyses: Correct choice rate

**Fig 4:**
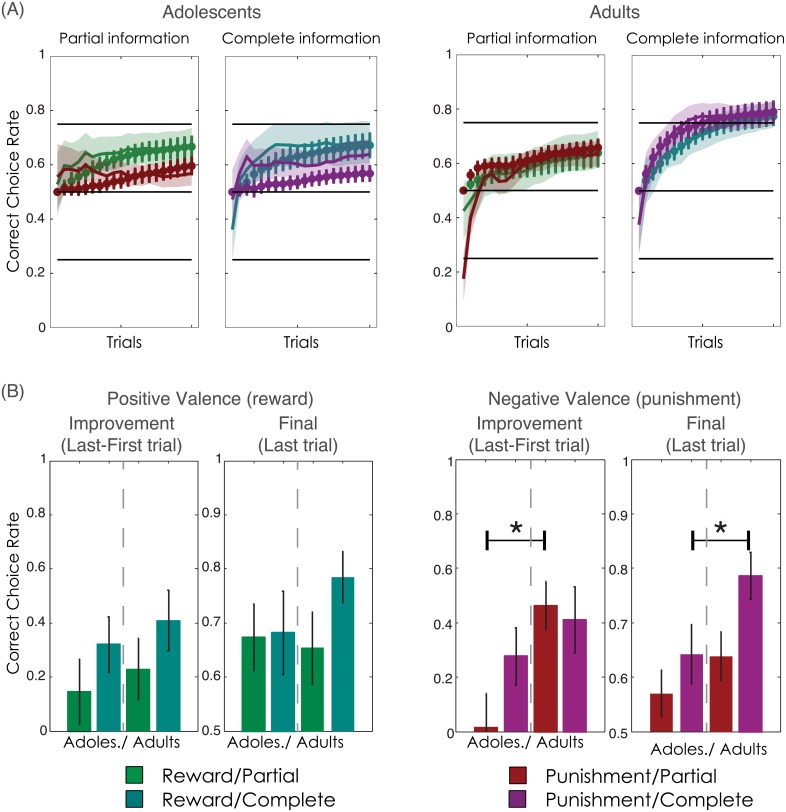
Correct choice rate. **(A)** Learning curves in adolescents (left) and adults (right). The bold lines within the shaded areas represent the actual behavioural data (bold lines represent mean correct choice rate; shaded areas represent s.e.m). The behavioural data are superimposed with the ex-post model-simulated learning curves, estimated using parameters from each age group’s best fitting model (Model 1 for adolescents; Model 3 for adults). The dots represent the model-simulated mean correct choice probabilities. (**B**) Bars represent the correct choice rate improvement (difference in correct choice rate between last and first trials) and the final correct choice rate (last trial) in Reward (leftmost panel) and Punishment (rightmost panel) contexts. Chance level (i.e. no learning) is 0.0 for correct choice rate improvement, and 0.5 for final correct choice rate. Error bars represent s.e.m. *P<0.05: independent samples t-test (2-sided).

Our model comparison analyses suggest that adults and adolescents do not use the same computational strategy (Fig 3B). If this is the case, this computational result should be reflected in behavioural differences between the two groups. To verify this, we analysed the correct choice rate learning curves using a mixed-design ANOVA with group (Adolescents vs. Adults) as the between-subjects factor and trial (1:20), valence (Reward vs. Punishment) and feedback information (Partial vs. Complete) as within-subjects factors (Fig 4A). There was a significant main effect of trial on correct choice rate (F(19,684) = 26.8, P<0.001), in which the rate of correct choices increased over the course of the learning task. There was also a significant interaction between group and trial (F(19,684) = 5.7 P<0.001), which was further moderated by valence (F(19,684) = 2.0, P<0.01). This suggests that adults and adolescents differed in the way their correct choice rate evolved during learning and that this difference interacted with outcome valence (Reward vs. Punishment). Post-hoc comparisons performed on the correct choice rate improvement (the difference between the first and last trials) indicated that, compared to adults, adolescents showed lower correct choice rate improvement in the Punishment/Partial context (T(36) = -2.9, P<0.01) (Fig 4B). Post-hoc comparisons performed on the correct choice rate in the final trial (trial 20) indicated that, compared to adults, adolescents had lower rates of correct choice in the Punishment/Complete context (T(36) = -2.1, P<0.05) (Fig 4B). Finally, while there was no significant interaction between feedback information and group, exploratory analyses indicated that whereas adults performed better in Complete feedback contexts (final correct choice rate: T(19) = 2.7, P<0.05), adolescents showed no such positive effect of counterfactual information on correct choice rate (T(17) = 0.9, P>0.4). To summarise, adolescents displayed reduced punishment learning compared to adults. Also consistent with our computational analyses, adolescent performance did not benefit from counterfactual feedback, although the interaction with group did not reach statistical significance (see Table 2).

**Table 2:** Behavioural data as function of choice context.

Dependent variables	Adolescents (N = 18)	Adults (N = 20)
**Correct choice rate (% correct)**		
Overall	64.1±5.0^#^	71.4±2.9^###^
Reward/Partial	67.4±6.2	65.3±6.8
Punishment/Partial	56.8±4.5	63.8±4.6
Reward/Complete	68.2±7.7	78.1±4.9
Punishment/Complete	63.8±5.6	78.6±4.3
**Reaction time (seconds)**		
Overall	0.79±0.03	0.83±0.03
Reward/Partial	0.78±0.03	0.78±0.03
Punishment/Partial	0.84±0.03	0.87±0.03
Reward/Complete	0.71±0.03	0.81±0.03
Punishment/Complete	0.82±0.03	0.85±0.03

Overall refers to average performance collapsed across contexts.

^#^P<0.05 and ^###^P<0.001 (2-sided, one-sample, t-test), when comparing to chance level—random—performance (i.e. 50% of correct responses).

Note that neither overall correct choice rate (T(36) = 1.3, P>0.1) nor overall reaction time (T(36) = 1.3, P>0.3) differed between groups (T(36) = 1.3, P>0.3).

### Ex-post model simulations: Learning task

The behavioural analyses support the model comparison analyses, suggesting that adolescents implement a simpler computational model than adults (Fig 4A and 4B). To further verify the ability of the models to reproduce the observed behaviour, we used the optimised model parameter values to simulate correct choice rate (ex-post model simulations; see Methods). Trial-by-trial model estimates of the probability of choosing the correct response in the learning task were generated for each participant using the best fitting model for their age group (i.e. Model 1 for adolescents; Model 3 for adults). Model-simulated data were submitted to the same analyses as the behavioural data, which indicated significant group x valence x trial (F(19,684) = 2.8, P<0.001), and group x feedback information x trial (F(19,684) = 8.7, P<0.001) interactions, consistent with the reduced capacity to learn from counterfactual information and to efficiently avoid punishments observed in adolescents (Fig 4A).

### Behavioural analyses: Reaction times

**Fig 5:**
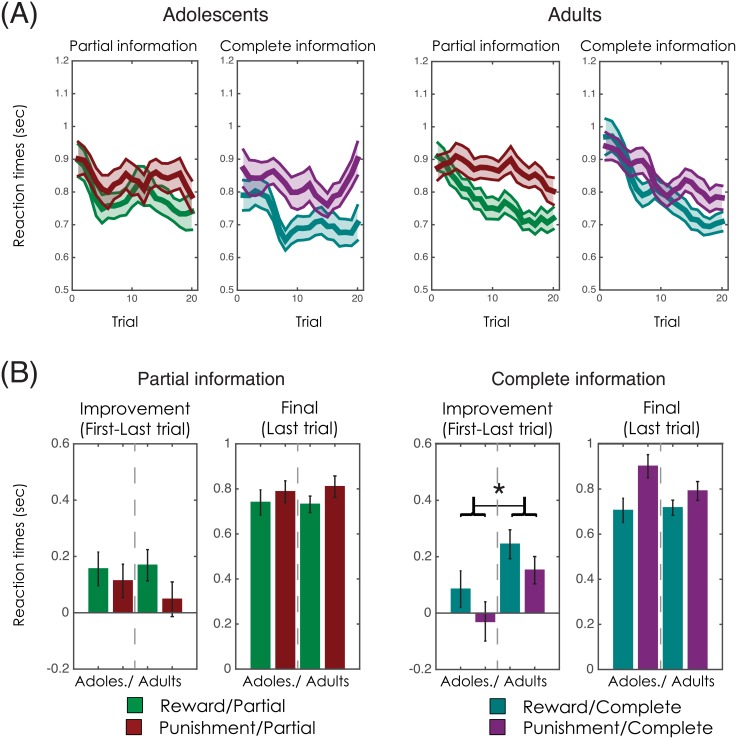
Reaction times. **(A)** Reaction time (RT) curves in adolescents (left) and adults (right). The bold lines within the shaded areas represent the mean RT. The shaded areas represent the s.e.m. (**B**) Bars represent the RT reduction (difference in RT between last and first trials) and the final RT (last trial) in the Partial (leftmost panel) and the Complete (rightmost panel) feedback contexts. Error bars represent the s.e.m. *P<0.05: independent-samples t-test (2-sided).

Although reinforcement learning models and paradigms are primarily concerned with choice data, RTs are also supposed to carry relevant information concerning both option and decision values[31,32]. RTs were analysed in the same way as correct choice rate. We analysed the RT curves with a mixed-design ANOVA with group (Adolescents vs. Adults) as between-subjects factor and trial (1:20), valence (Reward vs. Punishment) and feedback information (Partial vs. Complete) as within-subject factors (Fig 5). There was a significant main effect of trial on RT (F(19,684) = 12.1, P<0.001), reflecting a learning-induced RT reduction. There was also a significant main effect of valence (F(1,36) = 9.6, P<0.01), and a significant interaction between valence and trial (F(19,684) = 5.9, P<0.001), which reflected shorter RTs in the Reward compared to the Punishment contexts.

Post-hoc comparisons performed on the final RT reduction (RTs at trial 20) indicated that both adults and adolescents showed higher RT (i.e. slower responses) in the Punishment compared to the Reward contexts (adults: T(19) = 2.1, P<0.05; adolescents: T(17) = 2.9, P<0.05). We also found a significant interaction between feedback information and trial, indicating that RT reduction differed in Partial and Complete feedback contexts (F(19,684) = 2.3, P<0.001). There was no main effect of group on RT (F(1,36) = 1.6, P>0.2), however there was a significant interaction between group and feedback information (F(1,36) = 12.2, P<0.01), which was further moderated by trial (F(19,684) = 4.1, P<0.001), indicating that RT reduction in the two groups was differentially influenced by the presence of counterfactual information. Post-hoc comparisons performed on the RT reduction (i.e. RTs at trial 1 minus RTs at trial 20) indicated that, compared to adults, adolescents showed less of a reduction in RT in the Reward/Complete context, which was not quite significant (T(36) = 1.9, P<0.06) and the Punishment/Complete context, which was significant (T(36) = 2.2, P<0.05) (T(36) = 2.4, P<0.05; when collapsed across the two Complete contexts) (Fig 5B). Accordingly, whereas adult RT was reduced in the Complete compared to the Partial context (-89.8ms: T(19) = 2.4, P<0.05), adolescents increased their speed (+10.7ms; T(17) = 1.8, P<0.09). To summarise, in both age groups RTs are slower in the Punishment compared to the Reward contexts, which is consistent with an implicit Pavlovian inhibition effect[32]. Consistent with the model comparison analyses and choice, the influence of counterfactual information on RT over the course of the learning task was reduced in adolescents compared to adults (see Table 2).

### Behavioural analyses: Post-learning test

**Fig 6:**
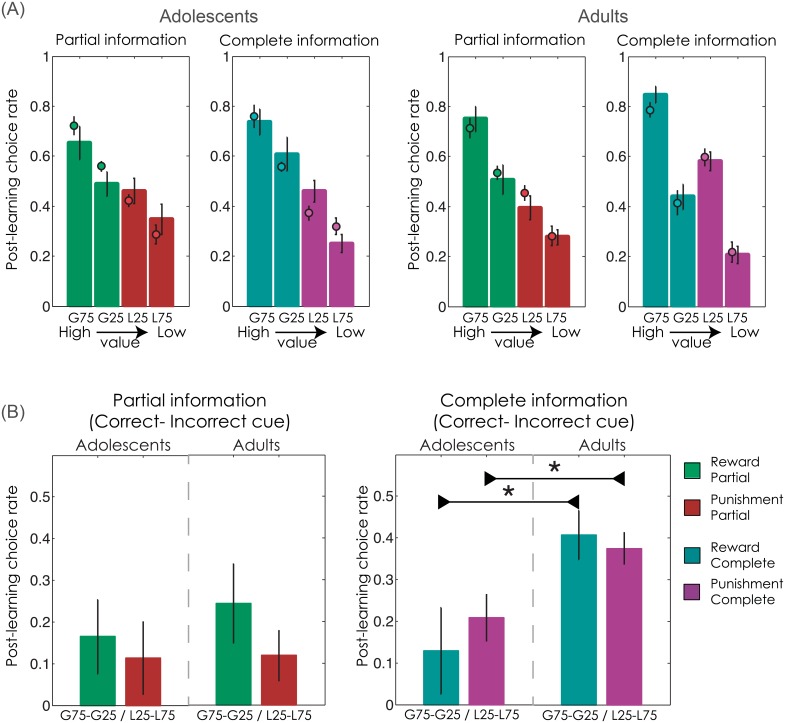
Post-learning test. **(A)** Bars represent the choice rate observed in the post-learning test, in adolescents (left) and adults (right). G75 and G25: options associated with 75% and 25% chance of gaining a point, respectively; L75 and L25: options associated with 75% and 25% chance of losing a point, respectively. The behavioural data are superimposed with coloured dots representing the model-simulated post-learning choices, estimated using parameters from each age group’s best fitting model (Model 1 for adolescents; Model 3 for adults). **(B)** Bars represent cue discrimination, the difference between post-learning choice-rates for Correct vs. Incorrect cues (G75 minus G25 in Reward contexts; L25 minus L75 in Punishment contexts), in Partial (leftmost panel) and Complete (rightmost panel) contexts. Chance level (i.e. no cue discrimination) is 0.0. Error bars represent s.e.m. *P<0.05: independent samples t-test (2-sided).

The post-learning test measured the ability to retrieve and transfer the value of the cues, as learnt by trial and error during the learning task. Post-learning choice rate was extracted for each of the eight cues and analysed using a mixed-design ANOVA with group (Adolescents vs. Adults) as a between-subjects factor, and cue valence (Reward vs. Punishment), feedback information (Partial vs. Complete), and cue correctness (Correct vs. Incorrect) as within-subject factors. There was a significant effect of valence (F(1,36) = 92.2, P<0.001) on post-learning choice rate, indicating that cues associated with Reward (G75 and G25) were preferred over those associated with Punishment (L25 and L75). Similarly, Correct cues (G75 and L25) were preferred over Incorrect ones (G25 and L75; F(1,36) = 38.1, P<0.001) (Fig 6). These effects indicate that, overall, participants were able to retrieve the value of the cues during the post-learning test. Crucially, the analysis also revealed a significant interaction between feedback information and cue correctness (F(1,36 = 11.6, P<0.01), which was further moderated by group (F(1,36 = 6.0, P<0.05). Post-hoc between-groups comparisons of these difference scores (Fig 6 and Table 3) indicated that cue discrimination was significantly lower in the adolescents than in the adults in both the Complete contexts (Reward/Complete: T(36) = -2.4, P<0.05; Punishment/Complete: T(36) = -2.6, P<0.05). While adults showed improved cue discrimination in Complete contexts compared to Partial contexts (T(19) = 4.1, P<0.001), adolescents did not (T(17) = 0.6, P>0.5). To summarise, in adults, cue value retrieval in the post-learning test was enhanced for cues associated with counterfactual feedback during the learning task. Adolescents did not show this effect.

**Table 3:** Post-learning test behavioural data as a function of cue type.

Post-learning choice rate	Adoles.	Adults
G_75_ Reward/Partial (% choices)	65.3±6.8	75.0±5.1
G_25_ Reward/Partial (% choices)	48.8±5.0	50.7±5.8
L_25_ Punishment/Partial (% choices)	46.0±5.0	39.5±4.7
L_75_ Punishment/Partial (% choices)	34.7±6.1	27.7±3.0
G_75_ Reward/Complete (% choices)	73.6±5.2	84.6±3.3
G_25_ Reward/Complete (% choices)	60.7±6.7	43.9±5.1
L_25_ Punishment/Complete (% choices)	45.8±4.3	58.0±3.8
L_75_ Punishment/Complete (% choices)	25.0±3.6	20.5±3.5

G75 and G25: options associated with 75% and 25% chance of gaining a point, respectively; L75 and L25: options associated with 75% and 25% chance of losing a point, respectively. Post-learning choice rates are reported as mean+-s.e.m.

### Ex-post model simulations: Post-learning test

We also tested the model’s ability to account for choices made in the post-learning test. Under the assumptions that choices in the post-learning test were dependent on the final option values in the learning task, and that there was no significant memory decay between the two tasks, the post-learning test, as in previous studies, can be used as an out-of-sample measure to compare the predictions of the different models[33,34] We calculated the probability of choice in the post-learning test using a softmax function, using the same individual choice inverse temperature optimized during the learning task (note that similar results have been obtained by optimising a beta specific to the post-learning test). Again, we submitted the model-simulated post-learning choice rates to the same statistical analyses as the behavioural data (Fig 6). Analysis of the model-simulated choices in the post-learning test also showed a significant group x feedback information x correctness interaction (F(1,36) = 13.0, P<0.001), consistent with the behavioural finding of enhanced cue value retrieval in adults for cues associated with counterfactual information that was not observed in adolescents, and the model comparison analyses. As indicated by the ex-ante model-simulated option values, higher cue discrimination in both the Reward/Complete and Punishment/Complete contexts and inverted preferences for intermediate value cues (i.e. small gains and small losses) requires both counterfactual learning and value contextualisation (Fig 2B).

## Discussion

Adolescents and adults performed an instrumental probabilistic learning task that involved learning to seek rewards or to avoid punishments. Feedback information was also manipulated: in some contexts, participants could only learn from the outcome of their choice, whereas in other contexts they could learn from both the outcome of the chosen and the unchosen option (counterfactual learning). Bayesian model selection indicated that a sophisticated model, incorporating a counterfactual learning module (necessary to learn from the unchosen option outcome) and a value contextualisation module (necessary to learn equally well from rewards and punishments) best accounted for adult behaviour, replicating previous findings[15]. Behavioural analyses showed that adults learnt equally well to seek rewards and avoid punishments and also efficiently integrated counterfactual information in instrumental learning. However, adolescent behaviour displayed a different pattern. In adolescents, Bayesian model selection significantly favoured the simplest action-value algorithm (Q-learning). This computational observation was supported by behavioural analyses of the learning task, in which the adolescents displayed reduced punishment avoidance learning, and in which RT reduction differed between adolescents and adults in the Reward/Complete context. Post-learning test analysis further corroborated the computational and behavioural findings of the learning test. Our findings support the hypothesis that adolescents and adults do not implement the same computational strategies.

### Reward learning

Within the factorial design of our task, the Reward/Partial context represented a “baseline” learning context. From a computational perspective, this context is the simplest as participants can efficiently maximise rewards by directly tracking outcome values using a basic model of reinforcement learning (RL). Neuroimaging and pharmacological studies have demonstrated the importance of subcortical structures, particularly the ventral striatum, in this basic reward-value learning[10,35]. The striatum shows earlier anatomical maturation compared with the more protracted development of the prefrontal cortex[24–26]. Basic reward seeking has also been associated with the dopaminergic modulation of the striatum[33,36,37], and animal studies show that striatal dopamine peaks during adolescence[38,39]. A previous task using a simple reward maximisation task, comparable to our Reward/Partial condition, showed stronger encoding of reward learning signals in the striatum in adolescents compared to adults, with no negative behavioural consequences [2]. Consistent with these data, we observed no differences between age groups in basic reward learning in the Reward/Partial context. The similar performance between groups in the Reward/Partial context provides evidence that the group differences we observed concerning punishment and reward learning cannot be explained by a generalised lack of motivation or attention, but rather are likely to be associated with specific computational differences.

### Counterfactual learning

While less extensively studied than simple action-value learning, previous neuroimaging and computational studies of counterfactual learning suggest that learning from the outcome of the unchosen option recruits dorsolateral and polar prefrontal structures[13,14,21]. We hypothesised that, since these regions are still developing in adolescence [40–43], adolescents would display a reduced ability to learn from counterfactual feedback. Both our computational and behavioural analyses (specifically the reaction times and post-learning test) supported this prediction. This reduced integration of counterfactual outcomes in adolescent behaviour is also consistent with a previous study showing limited feedback use as a possible source of higher risky decision-making during adolescents[44]. Counterfactual learning can also be understood within the framework of “model-based” (as opposite to “model-free”) RL[45,46]. Algorithms that operate without using a representation (model) of the environment, such as basic Q-learning, are termed model-free. Conversely, algorithms that build option values by simulating different possible courses of action (i.e. planning), based on an explicit model of the environment (the task), are termed model-based. Counterfactual learning can be conceptualised as a “model-based” process, as it involves the updating of option values according to mental simulations of what the outcome could have been if we had chosen an alternative course of action[21]. Like counterfactual learning, model-based learning has been theoretically and experimentally associated with prefrontal systems[47–49]. A key area for future research will be to examine whether or not the developmental changes in counterfactual learning observed here generalise to and interact with other forms of computation implicated in model-based learning, such as state transition learning.

### Punishment learning

In our task, symmetrical performance in the reward seeking and punishment avoidance learning conditions depends on the ability to contextualise outcome values. Value contextualisation consists of updating option value as a function of the difference between the experienced outcome and an approximation of the average value of the two options (i.e. the context value). Thus, in punishment contexts, where the overall context value is negative, an intrinsically neutral outcome (neither gaining nor losing points: 0pt; Figs 2A and 4) acquires a positive value and can therefore reinforce selection of the options that lead to successful avoidance of punishment. In the absence of value contextualisation, the neutral outcome, which represents the best possible outcome in the punishment contexts will inevitably be considered as less attractive than a positive outcome (the best possible outcome in the reward contexts: +1pt), and consequently the participant will perform less optimally in punishment contexts.

Previous studies of punishment avoidance learning, using the same or similar tasks as ours, have implicated the dorsomedial prefrontal cortex and dorsal anterior cingulate cortex in the representation of negative values and negative prediction errors[20,22]. Similarly to counterfactual learning, we predicted that adolescents would show reduced punishment avoidance learning based on the continuing development of prefrontal “control” regions. Indeed, our results demonstrated that adolescents were less likely to engage in value contextualisation computation and thus showed less effective punishment avoidance learning and different cue evaluation in the post-learning test. Thus, our results provide a computational substrate to neurobiological theories pointing to a reward/punishment imbalance as a driving force of adolescent risk- and novelty-seeking behaviour[6,24,26,50].

Previous studies of punishment avoidance learning in adolescents have elicited somewhat inconsistent results. While some studies showed a reduction of punishment learning in adolescents[51–53], others reported no effect of valence[54], or even higher performance in punishment than reward contexts[55,56]. One possible way to reconcile these discrepancies is to consider the modular nature of computational RL. In addition to value contextualisation, at least one other learning process, the Pavlovian inhibitory system, has been implicated in punishment avoidance learning[32]. According to this theory, and supported by experimental findings, Pavlovian expectations may influence choice behaviour via Pavlovian-Instrumental Transfer (PIT)[57]. In instrumental tasks, PIT is observed in the form of increased motor inertia for actions leading to potential harm (losses). Since Pavlovian learning has been shown to be underpinned by subcortical structures, such as the amygdala, which mature relatively early in adolescence [40,58], it is possible that PIT occurs similarly in adolescents and adults. We would predict that, for avoidance tasks that rely only on PIT, adolescents and adults would display similar performance, whereas in tasks that require value contextualisation (such as multi-armed bandit tasks, with probabilistic outcomes), adolescents and adults would not behave similarly. To investigate the Pavlovian inhibitory system in adolescent we considered the reaction time from stimulus onset to the decision point. We found that in both adolescents and adults, RTs were longer in Punishment than in Reward contexts. Interpreted within the framework of Pavlovian-Instrumental Transfer learning, this effect may reflect an increase in motor inertia of actions associated with potential losses. In other words, punishment avoidance actions require more time to be performed, compared to reward seeking actions, because avoidance is more naturally linked to “nogo” responses. It is possible that in adolescents the Pavlovian inhibitory system is fully responsive and can mediate successful punishment avoidance in tasks that do not require value contextualisation[55]. Finally, RT profiles differed between adolescents and adults in the Reward/Complete context, which may provide supplementary evidence of reduced counterfactual learning in adolescents. This “multiple systems” account of avoidance learning is also consistent with the proposal that reward/punishment imbalance in pathology, development and aging, could be underpinned by different neurophysiological mechanisms[59,60].

### Methodological implications

From a methodological perspective our study underlines the importance of using computational approaches to study the development of learning and decision-making[61,62]. Few studies have used computational models to interpret adolescent behaviour[63–65], and fewer still have implemented model comparison techniques[51,54]. Behavioural measures provide a relatively rough measure of performance in learning tasks for the following reasons. First, in probabilistic learning tasks an incorrect response, as defined by the experimenter with knowledge of the task design, may locally be a “correct” response, according to the actual history of choices and outcomes experienced by the participant, as a function of misleading trials. Second, the final estimation of learning performance may be affected by differences in initial choice rate. For example, a participant who starts choosing the correct option by chance is favoured compared to a participant who would need to “explore” the options in order to find out the correct option. Third, aggregate model-free analyses are not able to formally tease apart the possible computational processes underlying performance differences, which could be characterised either by differences in free parameter values within the same model, or by differences in the computational architecture itself. By incorporating into the analysis the individual history of choices and outcomes, and formalising different learning mechanisms in discrete algorithmic modules, computational model-based analyses offer an elegant solution to these issues. As such, our study, together with others, has be seen as part of a broader agenda aiming at moving from an “heuristic” to an “mechanistic” modelisation of human cognitive development[66].

### Conclusions and implications

Our results suggest that adolescents show heightened reward seeking compared to punishment avoidance learning and a reduced ability to take into account the outcomes of alternative courses of action. Together, these processes may contribute to the adolescent propensity to engage in value-based decision-making. Atypical value processing and learning are also implicated in multiple mental health disorders, at both the behavioural and neural level[67]. Increasing our understanding of normative changes in learning and decision-making during adolescence may thus provide insight into why adolescence is a period of increased risk for risky behaviours and mental health difficulties such as substance abuse and depression[68]. Finally, our results might also have implications for education, since they suggest that adolescents might benefit more from positive than from negative feedback when improving behavioural performance[69].

## Materials and Methods

### Participants

We recruited 50 volunteers aged between 12 and 32 years. Adolescents (N = 26; 12–17 years) were recruited from a local Community Theatre and UCL volunteer databases; adults (N = 24; 18–32 years) were recruited from UCL volunteer databases. The study was approved by the UCL Research Ethics Committee, and participants, or their legal guardians (adolescents), gave written informed consent. All participants were native English speakers and non-verbal IQ was assessed using the matrix reasoning subset of the Wechsler’s Abbreviated Scale of Intelligence (WASI)[70]. Due to group differences in non-verbal IQ scores (T(48) = 4.59, P<0.001), we restricted our analysis to those participants with scores falling within the range shared by both groups. The lower level of the range was determined by the lower IQ of the initial adult group, the higher IQ level of the range was determined by the higher IQ of the initial adolescent group. This gave a final sample of 38 participants, in which age groups (20 adults; 18 adolescents) were matched in non-verbal IQ and gender composition (see Table 4).

**Table 4:** Sample demographics.

Group	N	Age (years)	Gender	IQ (T-scores)
Adolescents	18	14.27 ± 0.30 (12–16)	8 male, 10 female	98.5 ± 1.1 (46–61)
Adults	20	22.35 ± 0.83 (18–32)	8 male, 12 female	101.4 ± 1.0 (43–61)

IQ: on-verbal IQ assessed using the matrix reasoning subset of WASI. Age and IQ are reported as mean+-s.e.m. IQ did not significantly differ ns between age groups: T(36) = 2.01, P>0.05.

All participants received a fixed amount of £5 for taking part, plus an additional amount (£0-£10) that varied according to their task performance (i.e. the average correct response rate). For “correct choice rate ≤ 0.50” participants received no bonus, for “0.50 > correct choice rate ≥ 0.75” participants received a £5 bonus, and for “correct choice rate > 0.75” participants received a £10 bonus. As a result of this payoff scheme, on average adults received £11.75±0.9 and adolescents £9.72±0.9 (payoff did not significantly differ between age groups: T(36) = 1.8, P>0.08).

Our group definition has, of course, limitations. We chose to define the adolescent group as individuals under the age of 18, and adults over the age of 18. This fits with societal definition of adulthood, but it is an inevitably arbitrary cut-off, and as such, it is possible that there might be developmental changes in task performance during in early adulthood that we cannot detect. The age range of the adolescent group is relatively large and, again, it is possible that developmental changes within this age range could be found.

### Behavioural task

Participants performed a probabilistic instrumental learning task adapted from a previous neuroimaging study [15]. The task had two phases, a learning task and a post-learning test. The learning task was designed to manipulate both outcome valence (Reward vs. Punishment) and feedback type (Partial vs. Complete; Fig 1) using a 2x2 factorial design. In the learning task, participants viewed pairs of abstract symbol cues (characters from the Agathodaimon alphabet) on a computer screen and had to choose one of the two. There were eight different cues, divided into four fixed pairs so that a given cue was always presented with the same counterpart. As such, the cue pairs represented stable choice contexts. Each of the four pairs corresponded to one of four context conditions (Reward/Partial, Reward/Complete, Punishment/Partial and Punishment/Complete). In Reward contexts, the ‘good’ outcome was gaining a point and a ‘bad’ outcome was not gaining a point, whereas in Punishment contexts, a ‘good’ outcome was not losing a point, while a ‘bad’ outcome was the loss of a point. Within each pair, one cue had a higher probability of resulting in a ‘good’ outcome (75%; the “correct” option; G75 and L25 cues) than the other (25%; the “incorrect” option; G25 and L75 cues). Depending on the pair of cues (i.e. choice context), participants were presented with only the outcome of the chosen cue (Partial feedback) or the outcomes of both the chosen and unchosen cues (Complete feedback). Each cue pair was presented 20 times in a pseudo-randomised order, giving a total of 80 trials. Cue pairs were presented either side of a central fixation cross, with side of presentation pseudo-randomised so that each cue was presented an equal number of times on each side.

Participants were instructed to acquire as many points as possible, as this would determine their final payment. We explained to participants that only their chosen outcome counted toward their points score, even if sometimes both outcomes were presented, and that both winning points and avoiding losing points were equally important to maximise payoff. After hearing the task instructions, participants performed a training session, before starting the learning task. Each trial started with a fixation cross (1 seconds), followed by presentation of the cue pairs (2 seconds), during which participants had to select either the left or right cue by pressing the corresponding button. After the choice window, a red arrow indicated the chosen option (0.5 seconds), before the cues disappeared and the chosen cue was replaced by the outcome (2 seconds; “+1pt” and a happy smiley, “0pt” and no image, or “-1pt” and unhappy smiley; Fig 1B). In Complete feedback contexts, the outcome corresponding to the unchosen option (counterfactual feedback) was also displayed. Note that while, on average, outcomes for each cue pair were anti-correlated on an individual trial, the outcomes of each cue were independent from one another. Thus, for example, in Complete feedback contexts participants could observe the same outcome for each cue (37.5% of trials).

After the learning task, participants completed a post-learning test of cue value. Here, the eight cues from the learning task were presented as unfixed pairs of all 28 possible pair-wise combinations[15,33,34]. Each pair was presented 4 times in a pseudo-random order, giving a total of 112 trials. For each cue pair, participants had to indicate the option with the highest value during the preceding learning session (i.e. the cue with the highest likelihood of resulting in a ‘good’ outcome). Unlike the learning task, choice was self-paced and no feedback was presented. Instructions for this task were given after the learning task, to prevent participants from explicitly memorising cue values. We informed participants that cues would not necessarily be shown in pairings that had been presented previously during the learning task. While participants could not earn points in this assessment, we encouraged participants to respond as if points were at stake.

### Behavioural analyses

From the learning task we extracted the correct choice rate and RT as the dependent variables. A correct response was defined as a choice directed toward the “good” stimulus (i.e., the most rewarding or the least punishing cue of the pair). Learning curves were computed from the trial-by-trial cumulative average of correct responses during the learning session. The cumulative average in a given trial “t” is calculated by averaging the correct choice rate from trial 1 to trial “t”. Statistical analyses were performed on the learning curves, using mixed-design ANOVA, with group (Adolescents vs. Adults) as the between-subjects factor, and trial (1:20), valence (Reward or Punishment) and feedback information (Partial or Complete), as within-subjects factors, and group (Adolescents or Adults) as the between-subjects factor. The “trial” factor is important to assess whether or not the effect are “learning-dependent”[71]. Between-group post-hoc comparisons were performed on final correct choice rate (which is directly proportional to the final number of points earned) and on the correct choice rate improvement (i.e. final minus initial correct choice rate at trial 20 minus correct choice rate at trial 1) using independent-samples t-tests. Examining both the final and the improvement in correct choice rate are important, if one is to draw conclusions regarding differences in learning. Reaction times were also extracted from the learning task, smoothed with a three trial sliding window and submitted to the same statistical model used for the correct choice rate. For RT, between-group post-hoc comparisons were performed on the RT reduction (i.e. RTs at trial 1 minus RTs at trial 20) and the final RT (RTs at trial 20).

Post-learning choice rate (i.e. the number of time a cues was chosen in the post-learning test divided by the number of trials the cue was presented in) indirectly reflects instrumental learning and should be higher for the more advantageous (“Correct”) cues of the learning task. Post-learning choice rate was extracted for each of the eight cues and analysed using a mixed-design ANOVA with group (Adolescents vs. Adults) as a between-subjects factor, and cue valence (Reward vs. Punishment), feedback information (Partial vs. Complete), and cue correctness (Correct vs. Incorrect) as within-subject factors. Between-group post-hoc comparisons were performed on the difference between Correct and Incorrect cues (i.e. G75 minus G25, in Reward contexts; L25 minus L75 in Punishment contexts) using independent samples t-tests (2-sided). This difference is a measure of cue discrimination: a significant and positive value indexes the participant’s tendency to prefer the optimal option during the preceding learning task.

Statistical analyses were performed using Matlab (www.mathworks.com) and R (www.r-project.org).

### Computational models

We analysed participants’ with reinforcement learning models [72]. 68 The goal of all models was to find the option that maximises the cumulative future reward (R) in each choice context (state: s). Our model space included three nested and increasingly sophisticated models (Fig 2A). Model 1 was a standard Q-learning model, which instantiates learning from direct experience by updating the value of the chosen option according to the outcome of each trial. Counterfactual information and the context in which choices are presented are not taken into account. In Model 2, the standard Q-learning model was augmented by a computational module enabling learning from counterfactual information[14]. Finally, in Model 3, Model 2 was further augmented by a contextual learning module, enabling the updating of option values relative to the choice context in which they were presented[73]. 69 Model 3, has recently been proposed to account for: i) the ability to perform similarity in both punishment and reward contexts; ii) counterfactual learning; and iii) inverted preferences for intermediate value cues (i.e. small gains and small losses) when assessed post-learning[15]. Model 3 updates option values in relation to the choice context in which they are presented. Since Model 1 and Model 2 can be considered as special cases of Model 3, we will describe only Model 3. We made a deliberate effort to keep these models as simple and parsimonious as possible. Model 3 tracks the mean of the distribution of values of the choice context and uses it to centre option values. Notably, this model represents a minimal departure from standard reinforcement learning algorithms that imply context or option values are updated with a delta rule, such as Q-learning and actor–critic algorithms[72]. Given our prior interest in the computational (dynamic) processes of learning, and also given that in our task we did not independently modulate outcome variance and valence, our model space did not include descriptive and aggregate economic models, such as cumulative prospect theory (CPT; [74]). Note, exploratory simulations showed that models with different learning rates for positive and negative prediction errors were not capable of discriminating between our task factors and predictions and were therefore not included (see S1 Text and S2 Fig).

At trial t the chosen (c) and the unchosen (u) option values of the current context (s) are updated with the Rescorla-Wagner rule (also called delta-rule)[12]:

$$Q_{t+1}{\left(s,c\right)}_{}=\;Q_{t}{\left(s,c\right)}_{}+\alpha_{1}\delta_{C,t}$$

and

$$Q_{t+1}{\left(s,u\right)}_{}=\;Q_{t}{\left(s,u\right)}_{}+\alpha_{2}\delta_{U,t},$$

The key idea behind Model 3 is that it separately learns and tracks the choice context value V(s). Crucially, the state value (V(s)) is not merely the sum of the option values, but rather it actively affects (controls) them. In fact V(s) is used to centre option prediction errors δ_C_ and δ_U_ as follows:

$$\delta_{C,t}=\;R_{C,t}–\;V\left(s\right)\;–\;Q_{t}\left(s,c\right)$$

and

$$\delta_{U,t}=\;R_{U,t}–\;V\left(s\right)\;–\;Q_{t}\left(s,u\right)$$

(in the Complete feedback contexts only, in the Partial feedback condition no counterfactual prediction error is calculated: *δ*_*U*,*t*_ *= 0*).

Consequently, the option values are no longer calculated on an absolute scale, but are relative to their choice context value *V(s)*. *V(s)* itself is learnt with a delta rule:

$$V_{t+1}{\left(s\right)}_{}=\;V_{t}{\left(s\right)}_{}+\alpha_{3}*\delta_{V,t},$$

where *α*_*3*_ is the context value learning rate and δ_V,t_ the context value prediction error, which is calculated as follows:

$$\delta_{V,t}=\;R_{Tot,t}–\;V_{t}\left(s\right),$$

where *R*_*Tot*_. is the average outcome of a trial and is calculated in the Complete feedback contexts as the average of the factual and the counterfactual outcomes as follows:

$$R_{Tot,t}=\;\left(R_{C,t}+\;R_{U,t}\right)\;/\;2.$$

Given that *R*_*Tot*_ is designed to be a measure that encompasses the value of both chosen and unchosen options, in order to incorporate the unchosen option in the Partial feedback trials we calculate *R*_*Tot*,*t*_ as follows:

$$R_{Tot,t}=\;\left(R_{C,t}+\;Q_{t}\left(s,u\right)\right)\;/\;2.$$

Model 2 can be derived from Model 3 by assuming no context value learning (*α*_*3*_ = 0). Model 1 can be derived from Model 2 by assuming no counterfactual learning (*α*_*2*_ = *α*_*3*_ = 0).

In all models decision-making relies on a softmax function. The probability of choosing the option ‘a’ over the option ‘b’ is given by:

$$P_{t}\left(s,a\right)\;=\;{\left(1\;+\;exp\left(\beta *\left(Q_{t}\left(s,b\right)\;–\;Q_{t}\left(s,a\right)\right)\right)\right)}^{-1},$$

where *β* is the inverse temperature parameter.

### Parameter optimisation and model selection procedure

In a first analysis, we optimised model parameters by minimising the negative log-likelihood of the data, given different parameter settings, using Matlab’s fmincon function initialised at different starting points, as described in [15] (ranges: 0<*β*<Infinite, and 0< *α*_*n*_<1). Note that model fitting and parameter optimisation involved the learning (and not the post-learning) data. Negative log-likelihoods and inverse temperature parameters (β) were used to compare the between-group baseline quality of fit (without taking into account the model complexity) (S1 Table). In a second analysis, we optimised model parameters by minimising the Laplace approximation to the model evidence (LPP):

LPP = log(ΣP(D|M,θ))),

where D, M and θ represent the data, model and model parameters, respectively (S2 Table). The LPP increases with the likelihood (a measure of quality of fit) and is penalised by the size of the parameter space (a measure of model complexity). Thus, the LPP represents a trade-off between accuracy and complexity and can guide model selection. In addition, LPP maximisation, by including priors over the parameters, avoids degenerate parameter estimates, due to the small number of trials and the noisiness of the data. To avoid bias in model selection the same priors were used for the adolescent and adult group. Individual LPPs were fed into the mbb-vb-toolbox (https://code.google.com/p/mbb-vb-toolbox/)[30], a procedure that estimates the expected frequencies and the exceedance probability for each model within a set of models, given the data gathered from all participants. Expected frequency is a quantification of the posterior probability of the model (denoted PP), i.e. the probability of the model generating the data obtained from any randomly selected participant. Exceedance probability (denoted XP) is the probability that a given model fits the data better than all other models in the set, i.e. has the highest PP. PP has an advantage over likelihood ratios as it can be directly compared between subjects (log likelihood ratios are calculated within subjects), which was necessary as our aim was to compare model fitting between age groups. Moreover, we validated the superior sensitivity of our model comparison procedure compared to the BIC using model simulation (see S2 Text). We submitted the negative log-likelihoods, the inverse temperature parameters (β) and the PP to a mixed-design ANOVA with group (Adolescents vs Adults) as the between-subjects factor and model (Models 1–3) as the within-subjects factor. Post-hoc comparisons (2-sided) were conducted using independent samples t-tests when comparing between groups, and one-sample t-tests when comparing within group and against chance-level. In a control analysis, we fitted the model to maximise the negative log-likelihood and the LPP, assuming a single set of parameters for each level (group-level optimisation) (see Tables 1, S1, S2 and S3).

### Model simulations

We performed both ex-ante and ex-post model simulations. Ex-ante model simulations, in which we simulated data from 1000 virtual participants, were used to illustrate the properties of each model. The parameter values used in these simulation were *β* = 5.0, *α*_*n*_ = 0.3, similar to values observed in previous studies [75,76]. Note that using different parameter values led to very similar results. For each model, we analysed the model estimates of the option values (Q(state, action)) and decision values (ΔQ(state) Fig 2B), both of which are associated with different aspects of task performance. In the learning task, performance is a function of the learned difference in Q-values (ΔQ(state)) between the correct and incorrect option (decision value); in contrast, preference in the post-learning test allows inferences to be made about the value of individual options, which cannot be directly inferred from learning performance. Ex-ante model simulations were not submitted to statistical testing because the “N” is arbitrary.

Once we had optimised the model parameters, we used ex-post model simulations of the data to assess their generative performance by analysing the model simulation of the data[77] (“ex-post” model simulations). Model estimates of choice probability were generated on a trial-by-trial basis using the individual history of choices and outcomes. For each participant, we used their best fitting set of model parameters from their age group’s best fitting model (i.e. Model 1 for adolescents; Model 3 for adults). Model-simulated correct choice probability was then submitted to the same statistical analysis that was used to assess the actual choices made by participants in the learning task. Note that qualitative discrepancies between actual and simulated data at the beginning of the learning curve should be interpreted with caution. In fact, in the behavioural data the variance is higher in the early trials and then progressively decreases due to integrating over the past trials, whereas in model simulations the variance follows a different trajectory. By definition, the variance is zero in the first trial, in which the probability of a correct response is 0.5 for all virtual participants/contexts and then progressively increases following individual histories of choice and outcomes, as well as individual differences in free parameters.

## Supporting Information

S1 Text Supplementary model comparison: Value contextualisation without counterfactual learning. (DOCX)

S2 Text Supplementary model simulations (I): Validation of the model comparison procedure. (DOCX)

S3 Text Supplementary model simulations (II): Different learning rates for positive and negative prediction errors. (DOCX)

S1 Table Negative log-likelihood maximisation. Random: random model that assumes chance performance for all trials; p(correct choice) = 0.5. Subject-level: parameter optimization assumes a set of free parameters per subject. Group-level: parameter optimisation assumes a single set of free parameters per age group.
(DOCX)

S2 Table Model parameters. Parameters were optimised by minimising the Laplace approximation to the model evidence (LPP). Note that the group-level, adolescents were systematically fitted with α2 = α3 = 0 (basic Q-learning), whereas adults were fitted with α2>0 and α3>0 whenever the model allowed the parameter to be different from zero. Data are reported as mean±s.e.m. β: inverse temperature; α1: factual learning rate; α2: counterfactual learning rate; α3: contextual learning rate. Subject-level: parameter optimisation assumes a set of free parameters per subject. Group-level: parameter optimisation assumes a single set of free parameters per age group.
(DOCX)

S3 Table Log-likelihood differences. For each pair of models we calculated the log likelihood difference multiplied by 2, which is a log-scale analogue of the likelihood ratio. M1 to M3: Model 1 to 3. M0: the random model. Subject-level: parameter optimisation assumes a set of free parameters per subject. Group-level: parameter optimisation assumes a single set of free parameters per age group. “Adoles. % of Adults”: indicates the percentage of likelihood difference improvement observed in the Adolescent group compared to the Adult group (when accounting for the different number of subjects).
(DOCX)

S4 Table Supplementary model comparison. PP: posterior probability. XP: exceedance probability. Model 1: α_2_ = α_3_ = 0; Model 2: α_3_ = 0; Model 4: α_2_ = 0. PP are reported as mean±s.e.m.
(DOCX)

S1 Fig Validation of the model comparison procedure. **(A**) Virtual groups’ parameters and simulated data, plotted as a function of task context. **(B)** Possible and obtained model comparison results for Group 1 (light grey) and Group 2 (dark grey): better fit is indicated by a higher PP and lower BIC, respectively.
(EPS)

S2 Fig Model simulation involving different learning rates for positive and negative prediction errors. The value of the β was 5.0 as in the other ex-ante model simulations. The number of virtual participants was N = 1000.
(EPS)
