# Supplementary material for: The Computational Development of Reinforcement Learning during Adolescence
Source: PLoS Comput Biol. 2016 Jun 20;12(6):e1004953. doi: 10.1371/journal.pcbi.1004953 (PMC4920542; doi:10.1371/journal.pcbi.1004953)
Supplement: S1 Text — (DOCX) [file pcbi.1004953.s001.docx]

**Supplementary model comparison: value contextualisation without counterfactual learning**

Our model space did not include a model in which standard Q-learning (Model 1) was augmented with value contextualisation (α3>0) but not counterfactual learning (α2=0). The first reason for this decision was “historical”: previous studies involving counterfactual feedback have already proposed models implementing counterfactual learning. Thus, the counterfactual learning module did not represent a novel approach, but rather a benchmark by which to compare to the value contextualisation module(*1*–*3*). The second reason was conceptual: since the counterfactual learning module learns value in a policy-independent manner (i.e. independent from participants’ actual choices), its update rule in the Complete feedback conditions requires the integration of information from both the chosen (R_C_) and the unchosen outcomes (R_U_):

*δ_V,t_ = (R_C,t_ + R_U,t_) / 2. – V_t_(s)*.

Accordingly, we believe that a model that integrates (R_U_) information to update context value but does not use this information to update the unchosen option value would be conceptually unsound. However, for the sake of completeness, we nonetheless ran an additional model comparison analysis involving such a model (Model 4; value contextualisation without counterfactual learning) and found that the addition of this model did not affect our main results and conclusions: adolescent behaviour was still better explained by Model 1 (Model 1: PP=0.49±0.04; XP=0.6; Model 4 PP=0.08±0.02; XP=0) and adult behaviour was better explained by Model 3 (Model 3: PP=0.49±0.04; XP=0.6; Model 4 PP=0.08±0.02; XP=0) (**S4 Table**).

**References**

1. A. G. Fischer, M. Ullsperger, *Neuron*. **79**, 1243–55 (2013).

2. E. D. Boorman, T. E. Behrens, M. F. Rushworth, *PLoS Biol.* **9** (2011), doi:10.1371/journal.pbio.1001093.

3. J. Li, N. D. Daw, *J. Neurosci.* **31**, 5504–5511 (2011).
