# Supplementary material for: The Computational Development of Reinforcement Learning during Adolescence
Source: PLoS Comput Biol. 2016 Jun 20;12(6):e1004953. doi: 10.1371/journal.pcbi.1004953 (PMC4920542; doi:10.1371/journal.pcbi.1004953)
Supplement: S2 Text — (DOCX) [file pcbi.1004953.s002.docx]

**Supplementary model simulations (I): validation of the model comparison procedure**

Bayesian model comparison methods are highly debated in cognitive neuroscience(*1*, *2*). Different model comparison criteria have been proposed, which approximate model evidence while representing a trade-off between model accuracy and complexity. Critically, based on the same data, different criteria might provide different answers, since sample size, number of observation, the size of the effect to be detected and the presence of outliers differentially affects model comparison results(*3*). A priori model simulations can be used to assess the adequacy of model comparison criterion for a given dataset a priori. We consider a model comparison criterion “adequate” when 1) it correctly rejects a more complex model, when the true generative model is the simpler one (i.e. it avoids “overfitting") and 2) it correctly rejects a simpler model, when the true generative model is a more complex one (i.e. it avoids “underfitting”). We applied these principles to our study, by testing the adequacy of the frequently used Bayesian Information Criterion (BIC)(*4*) and the more recently introduced Laplace approximation to the model evidence (LPP), from which model posterior probability (PP) and exceedance probability (XP) can be computed (see **Methods**)(*5*, *6*).

We simulated data from two groups of virtual participants (N=200; **S1 Figure A**). Group 1 implemented Model 1 (standard Q-learning, with only two free-parameters (β, α1), whereas Group 2 implemented Model 3 (the more sophisticated model: standard Q-learning with two additional free-parameters accounting for counterfactual learning (α2) and value contextualisation (α3)). The parameter values in both groups were similar to those used in the model simulations presented in the main text (**Methods: Model simulations)**. Both groups of participants performed our learning task and produced very different behavioural results: Group 1 displayed preferential reward seeking compared to punishment avoidance learning and no performance enhancement in presence of counterfactual information, as expected from Model 1-estimates of behaviour (see **Fig. 2B**); Group 2 displayed similar learning from rewards and punishments and a performance enhancement in presence of counterfactual information, as expected from a Model 3-estimates of behaviour. For each virtual participant, we optimised model parameters by minimising the negative log-likelihood of the data, to calculate the BIC, and minimising the LPP, to calculate the PP. The BIC criterion correctly rejected Model 3 in Group 1, however failed to identify Model 3 as the correct model in Group 2 (Model 2 was selected as the best fitting model). In contrast, the LPP-based PP criterion correctly rejected Model 3 in Group 1 and correctly selected Model 3 in Group 2 (**S1 Figure B**). These results clearly indicate that for our task design and model space the LPP-based calculation of the PP is an “adequate” model comparison criterion, while the BIC is not.

This result is differs to our previous study in adults(*7*), in which both the BIC and LPP analyses detected Model 3 as the wining model. This may be due to several reasons. Here we use a modified version of the task used in the previous study, with a reduced number of trials and sessions (due to practical constraints of working with developmental populations), resulting in a smaller and potentially noisier dataset. Furthermore, while Model 2 and 3 produce crucial differences in behavioural patterns, the likelihood gain when moving from Model 2 to Model 3 is smaller than that between Model 1 to Model 2 (**Fig. 2** & **S1 Table**, **S2 Table** and **S3 Table**). We therefore conclude that the LPP-based criterion is the most appropriate model selection criteria for our dataset and model set and is capable of providing reliable results that are in line with the quantitative and qualitative behavioural differences observed in both our simulated and previous data(*7*).

**References**

1. M. a. Pitt, I. J. Myung, *Trends Cogn. Sci.* 6, 421–425 (2002).

2. G. S. Corrado, L. P. Sugrue, J. R. Brown, W. T. Newsome, in *Neuroeconomics: Decision Making and the Brain*, P. W. Glimcher, E. Fehr, C. F. Camerer, R. a Poldrack, Eds. (Academic Press, London, UK, ed. 2009, 2009), pp. 463–480.

3. K. E. Stephan, W. D. Penny, J. Daunizeau, R. J. Moran, K. J. Friston, *Neuroimage*. 46, 1004–1017 (2009).

4. Schwarz G, *Ann. Stat.* (1978).

5. N. D. D. Daw, S. J. J. Gershman, B. Seymour, P. Dayan, R. J. J. Dolan, *Neuron*. 69, 1204–1215 (2011).

6. J. Daunizeau, V. Adam, L. Rigoux, *PLoS Comput. Biol.* 10, e1003441 (2014).

7. S. Palminteri, M. Khamassi, M. Joffily, G. Coricelli, *Nat. Commun.* 6, 8096 (2015).
