# Supplementary material for: The Computational Development of Reinforcement Learning during Adolescence
Source: PLoS Comput Biol. 2016 Jun 20;12(6):e1004953. doi: 10.1371/journal.pcbi.1004953 (PMC4920542; doi:10.1371/journal.pcbi.1004953)
Supplement: S3 Text — (DOCX) [file pcbi.1004953.s003.docx]

**Supplementary model simulations (II): different learning rates for positive and negative prediction errors**

In principle, differences in performance between reward seeking and punishment avoidance domains could arise from the presence of different learning rates for positive prediction errors (frequently associated with rewards: α+) and negative prediction errors (frequently associated with punishments: α-)(*1*). Thus, it could be argued that asymmetrical performance in reward seeking and punishment avoidance learning conditions, as predicted by Model 1 (reward > punishment learning), results from differential learning rates, whereby the negative learning rate is higher than the positive learning rate.

However, in our task, in which the outcomes are probabilistic and the Reward and Punishment contexts are separated (i.e. a reward, +1pt, never occurs in the Punishment context and a punishment, -1pt, never occurs in the reward context), positive and negative prediction errors can occur in both the reward and punishment contexts. Instead, symmetrical performance in the reward seeking and punishment avoidance learning conditions (as predicted by Model 3) depends on the ability to contextualise outcome values (as a function of the difference between the experienced outcome and the context value; an approximation of the average value of the two options), whereby in a negative value context, an intrinsically neutral outcome (0pt) can acquire a positive value and reinforce selection of the options that lead to successful avoidance of punishment. In the absence of value contextualisation, whereas the optimal outcome in the Reward contexts, 1pt results in a positive prediction error, the optimal outcome in the Punishment contexts, 0pt remains intrinsically neutral in value and consequently the participant will perform less optimally in punishment contexts, independently of whether learning rates differ between positive and negative prediction errors.

To demonstrate this, we ran additional simulations for a model with higher learning rates for positive compared to negative prediction errors (αα), another with higher learning rates for negative compared to positive prediction errors (αα) and compared the simulated variables with those from a model with symmetrical learning rates (our Model 1: αα) (**S2** **Figure**). For these simulations we used learning parameters ranges comparable to those observed in previous studies (*2*). Model simulations showed that (at least in the range of parameters tested) the model with (α>α) is still not capable of explaining symmetrical performance in the reward and punishment domain. We also note that neither model is structurally capable of explaining option value inversion (as observed in the post-learning test).

**References**

1. Y. Niv, J. J. a Edlund, P. Dayan, J. J. P. O’Doherty, J. P. O. Doherty, *J. Neurosci.* **32**, 551–562 (2012).

2. G. Lefebvre, M. Lebreton, F. Meyniel, S. Bourgeois-Gironde, S. Palminteri, “Asymmetric reinforcement learning: computational and neural bases of positive life orientation” (Biorxive, 2016), , doi:10.1101/038778.
