# Supplementary material for: The Computational Development of Reinforcement Learning during Adolescence
Source: PLoS Comput Biol. 2016 Jun 20;12(6):e1004953. doi: 10.1371/journal.pcbi.1004953 (PMC4920542; doi:10.1371/journal.pcbi.1004953)
Supplement: S1 Table — Random: random model that assumes chance performance for all trials; p(correct choice) = 0.5. Subject-level: parameter optimization assumes a set of free parameters per subject. Group-level: parameter optimisation assumes a single set of free parameters per age group. (DOCX) [file pcbi.1004953.s004.docx]

|  | | **Random** | **Model 1** | **Model 2** | **Model 3** |
| --- | --- | --- | --- | --- | --- |
| **Subject -level** | **Adoles.** | 998.1 | 817.1 | 772.6 | 771.2 |
|  | **Adults** | 1109.0 | 865.2 | 752.3 | 733.1 |
| **Group- level** | **Adoles.** | 998.1 | 922.9 | 922.9 | 922.9 |
|  | **Adults** | 1109.0 | 962.2 | 897.2 | 895.9 |
