# Supplementary material for: The Computational Development of Reinforcement Learning during Adolescence
Source: PLoS Comput Biol. 2016 Jun 20;12(6):e1004953. doi: 10.1371/journal.pcbi.1004953 (PMC4920542; doi:10.1371/journal.pcbi.1004953)
Supplement: S2 Table — Parameters were optimised by minimising the Laplace approximation to the model evidence (LPP). Note that the group-level, adolescents were systematically fitted with α2 = α3 = 0 (basic Q-learning), whereas adults were fitted with α2>0 and α3>0 whenever the model allowed the parameter to be different from zero. Data are reported as mean±s.e.m. β: inverse temperature; α1: factual learning rate; α2: counterfactual learning rate; α3: contextual learning rate. Subject-level: parameter optimisation assumes a set of free parameters per subject. Group-level: parameter optimisation assumes a single set of free parameters per age group. (DOCX) [file pcbi.1004953.s005.docx]

|  |  | **Model 1** | | **Model 3** | | | **Model 3** | | | |
| --- | --- | --- | --- | --- | --- | --- | --- | --- | --- | --- |
|  |  | **β** | **α1** | **β** | **α1** | **α2** | **β** | **α1** | **α2** | **α2** |
| **Subject-level** | **Adoles.** | 4.18±0.91 | 0.45±0.06 | 5.19±1.22 | 0.36±0.05 | 0.29±0.05 | 5.20±1.39 | 0.33±0.04 | 0.38±0.05 | 0.42±0.06 |
|  | **Adults** | 4.66±0.81 | 0.49±0.06 | 7.85±1.28 | 0.36±0.05 | 0.29±0.04 | 8.65±1.45 | 0.35±0.06 | 0.28±0.04 | 0.39±0.07 |
| **Group-level** | **Adoles.** | 2.73 | 0.38 | 2.73 | 0.38 | 0.00 | 2.73 | 0.38 | 0.00 | 0.00 |
|  | **Adults** | 3.90 | 0.39 | 5.69 | 0.24 | 0.23 | 6.09 | 0.21 | 0.21 | 0.04 |
