# Supplementary material for: The Computational Development of Reinforcement Learning during Adolescence
Source: PLoS Comput Biol. 2016 Jun 20;12(6):e1004953. doi: 10.1371/journal.pcbi.1004953 (PMC4920542; doi:10.1371/journal.pcbi.1004953)
Supplement: S3 Table — For each pair of models we calculated the log likelihood difference multiplied by 2, which is a log-scale analogue of the likelihood ratio. M1 to M3: Model 1 to 3. M0: the random model. Subject-level: parameter optimisation assumes a set of free parameters per subject. Group-level: parameter optimisation assumes a single set of free parameters per age group. “Adoles. % of Adults”: indicates the percentage of likelihood difference improvement observed in the Adolescent group compared to the Adult group (when accounting for the different number of subjects). (DOCX) [file pcbi.1004953.s006.docx]

|  | | **M1 vs. M0** | **M2 vs. M1** | **M3 vs. M2** |
| --- | --- | --- | --- | --- |
| **Subject -level** | **Adoles.** | 362.0 | 88.9 | 2.8 |
|  | **Adults** | 487.5 | 225.8 | 38.6 |
| **Adoles. % of Adults** | | 82.5% | 43.7% | 8.0% |
| **Group- level** | **Adoles.** | 150.4 | 0 | 0 |
|  | **Adults** | 293.7 | 130.0 | 2.4 |
| **Adoles. % of Adults** | | 56.9% | 0% | 0% |
