# Supplementary material for: The Computational Development of Reinforcement Learning during Adolescence
Source: PLoS Comput Biol. 2016 Jun 20;12(6):e1004953. doi: 10.1371/journal.pcbi.1004953 (PMC4920542; doi:10.1371/journal.pcbi.1004953)
Supplement: S4 Table — PP: posterior probability. XP: exceedance probability. Model 1: α2 = α3 = 0; Model 2: α3 = 0; Model 4: α2 = 0. PP are reported as mean±s.e.m. (DOCX) [file pcbi.1004953.s007.docx]

|  | |  | **Model 1 (2df)** | | **Model 2 (3df)** | | **Model 3 (4df)** | | **Model 4 (3df)** | |
| --- | --- | --- | --- | --- | --- | --- | --- | --- | --- | --- |
|  | |  | **PP** | **XP** | **PP** | **XP** | **PP** | **XP** | **PP** | **XP** |
|  | **Adoles.** | | 0.43±0.05 | 0.73 | 0.19±0.03 | 0.07 | 0.12±0.02 | 0.03 | 0.26±0.05 | 0.17 |
|  | **Adults** | | 0.03±0.01 | 0.00 | 0.39±0.04 | 0.31 | 0.49±0.04 | 0.69 | 0.08±0.02 | 0.00 |
